# Characterization of the complete chloroplast genome of ‘Yunning No.1’ lemon (*Citrus limon*)

**DOI:** 10.1080/23802359.2020.1870889

**Published:** 2021-02-09

**Authors:** Shao-hua Wang, Guang-zhou Deng, Jun-yan Gao, Hong-ming Liu, Xian-yan Zhou, Jian-qiang Yue, Mei-chao Dong, Fan Yang, Dong-guo Zhou, Li-na Guo, Xiao-hui Yuan, Chun-rui Long

**Affiliations:** a Yunnan Academy of Agricultural Sciences, Institute of Tropical and Subtropical Cash Crops, Baoshan, PR China; b Guangxi Academy of Specialty Crops, Guilin, PR China; c Longling Hengguan Teda Agricultural Development co., LTD, Baoshan, PR China

**Keywords:** Lemon, Chloroplast genome, Phylogenetic analysis

## Abstract

‘Yunning No.1’ lemon, a mutant of Eureka lemon, is originally found in Yunnan province of China and is the main cultivated lemon variety there. In this study, we assembled and annotated its chloroplast genome using Illumina Hiseq-2500 whole genome re-sequencing data. Its chloroplast genome is 160,141 bp in size, containing a 87,754 bp large single copy region, a 18,385 bp small single copy region and a pair of 27,001 bp inverted repeat region. Like many citrus species, 114 unique genes (including 80 protein-coding genes, 30 tRNAs and 4 rRNAs) could be identified from the chloroplast genome of ‘Yunning No.1’. Phylogenetic analysis revealed that the ‘Yunning No.1’ chloroplast genome was closest to *Citrus maxima*.

‘Yunning No.1’ lemon (*Citrus limon* cv. ‘Yunning No.1’), a Eureka lemon bud mutant, is originally found in Dehong state, Yunnan province of China and is now the main lemon variety there. Nowadays, ‘Yunning No.’ lemon is mainly cultivated in tropic and subtropic areas of Yunnan province with sporadic cultivation in Hainan, Sichuan, Guangxi and Guangdong provinces of China. Its total planting area is more than 3000 hectares, among which more than 2000 hectares are in Yunnan province (Gao et al. 2014). Recently studies on ‘Yunning No.1’ were mainly focused on morphological phenotype, flowers, fruit and so on (Gao et al. 2014; Li et al. 2017), buy researches on its genetic information is very limited. In the present study, we assembled and annotated its complete chloroplast genome, based on which its phylogenetic relationships with other citrus species were also investigated.

The specimen of ‘Yunning No.1’ was collected from the citrus germplasm resource garden of the Institute of Tropical and Subtropical Cash Crops, Yunnan Academy of Agricultural Sciences, Baoshan city, Yunnan province, China (23°24′23.40″N; 102°03′50.35″E) and samples were deposited at Institute of Tropical and Subtropical Cash Crops, Yunnan Academy of Agricultural Sciences. The leaf genomic DNA of ‘Yunning No. 1’ was isolated using the CTAB method (Xie et al. 2020) and stored at the Institute of Tropical and Subtropical Cash Crops, Yunnan Academy of Agricultural Sciences (Code number YN01). The whole genomic DNA re-sequencing was performed on the Illumina Hiseq-2500 platform to generate 125 bp pair end reads (BIG, Shenzhen, CA, CHN). Totally, we obtained about 15.58 Gbp high quality clean reads, which were aligned to chloroplast genomes of six citrus species, including *C. maxima* (NC_034290.1), *C. sinensis* (DQ864733.1), *C. aurantiifolia* (KJ865401.1), *C. platymamma* (NC_030194.4), *Citrus limon* (KY085897.1), *C. depressa* (LC147381.1) and *C. reticulata* (NC_034671.1), assembled into chloroplast genome and annotated according to our previous studies (Zhang et al. 2020a, 2020b). The annotated chloroplast genome has been deposited in Genbank with the accession number MT880608.

The complete chloroplast genome of ‘Yunning No.1’ is 160,141 bp in size, containing a large single copy region of 87,754 bp, a small single copy region of 18,385 bp, and a pair of inverted repeat regions of 27,001 bp. Sequence annotation identified 114 unique genes (including 80 protein-coding genes, 30 tRNA genes and 4 rRNA genes) from the chloroplast genome of ‘Yunning No.1’. Similar to the *Citrus cavaleriei* chloroplast genome (Zhang et al. 2020a), nine protein coding genes (i.e. *ndhB*, *rpl2*, *rpl22*, *rpl23*, *rps7*, *rps12*, *rps19*, *ycf2* and *ycf15*), seven tRNA genes (i.e. *trnA-UGC*, *trnI-CAU, trnI-GAU*, *trnL-CAA*, *trnN-GUU*, *trnR-ACG* and *trnV-GAC*) and all the 4 rRNA genes (*rrn4.5*, *rrn5*, *rrn16* and *rrn23* rRNAs) were found to be occur in double copies. The overall nucleotide composition of the chloroplast genome is: 30.47% A, 31.05% T, 19.61% C, and 18.87% G, with the total GC content of 38.48%.

By using the complete chloroplast genomes of Yunning No.1 and 21 other plant species including 20 plants species from Aurantioidae, Rutoideae and Toddalioideae subfamilies and *Ailanthus altissima* (as outgroup), a maximum-likelihood phylogenetic tree was constructed to reveal its phylogenetic relationship with *Citrus* species from the chloroplast genome level. Surprisingly, results showed that the chloroplast sequence relationship between ‘Yunning No.1’ and *Citrus maxima*, rather than ‘Yunning No.1’ and *Citrus limon* (KY085897), was the closest (Figure 1). By checking the paper about the *Citrus limon* (KY085897) (Camarda et al. 2013), we found that it is named as *Citrus limon* var. pompia Camarda, but its fruits differ greatly from lemon fruits, indicating that it may not belong to lemon. And in the study of Wu et al. (2018), pummelo (*Citrus maxima*) is reported to be one of the ancestors of lemon. Thus, the closest relationship found between ‘Yunning No.1’ and *Citrus maxima* is more believable.

**Figure 1.**
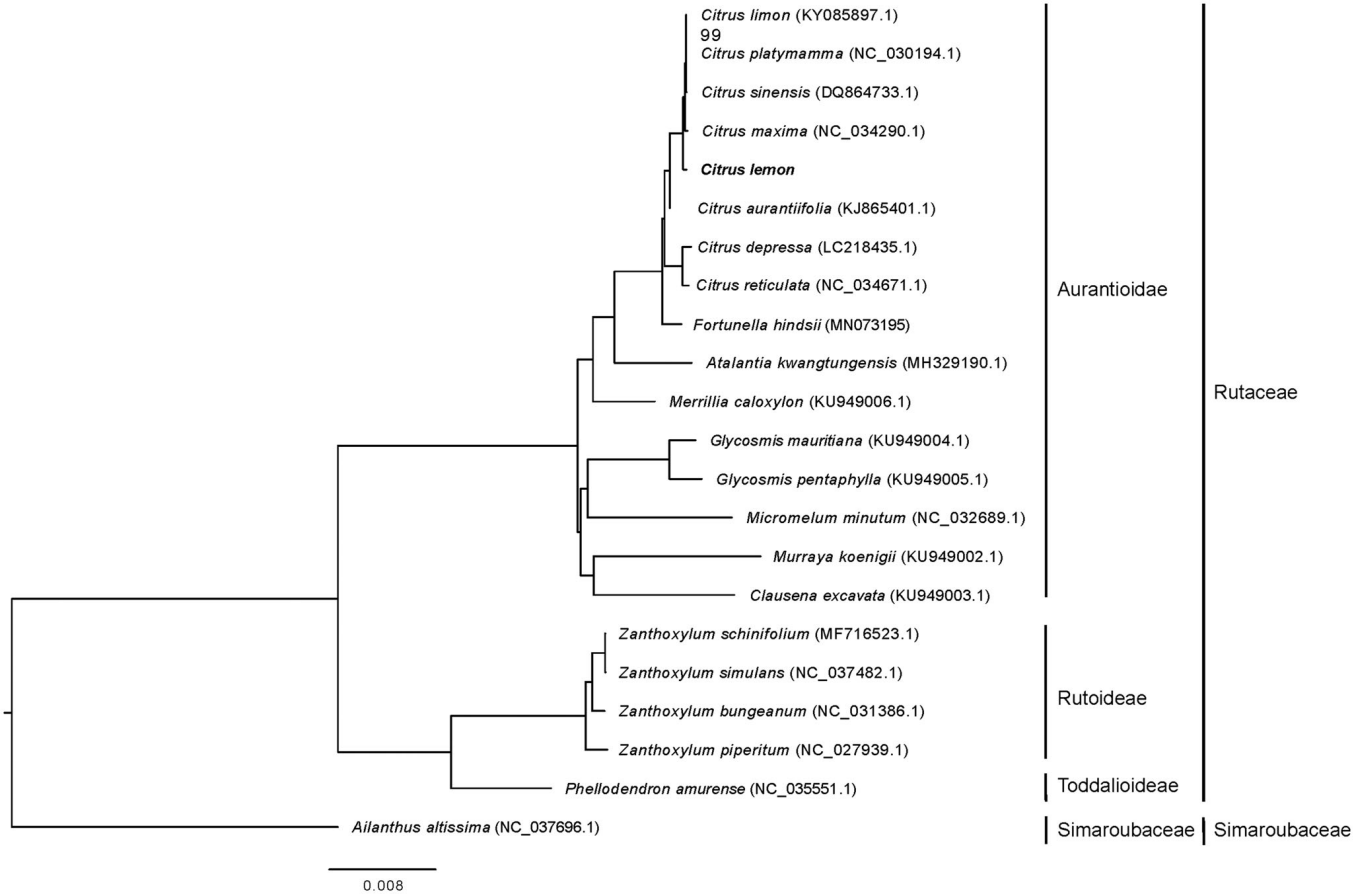
The maximum likelihood phylogenetic tree constructed using the complete chloroplast genome sequences of ‘Yunning No.1’ and 20 Rutaceae species. Numbers near the nodes are bootstrap value based on 1000 replicates, bootstrap values of 100 were omitted.

## Data Availability

The raw data that support the findings of this study is openly available in NCBI Sequence Read Archive at [https://www.ncbi.nlm.nih.gov/bioproject/PRJNA660156] under the BioProject ID PRJNA660156 and the annotated chloroplast genome has been deposited in Genbank [https://www.ncbi.nlm.nih.gov/genbank/] under the reference number MT880608.
